# The Time In Between: A Memoir of Hunger and Hope

**DOI:** 10.1192/pb.bp.115.051029

**Published:** 2016-04

**Authors:** Jane Morris

I was set to condemn yet another narcissistic autobiographical account that would be pro-anorexia fodder – but the author herself cleverly forestalls that objection. She acknowledges that we can't stop ‘wannarexics’ from devouring this as a motivational manual. Thus from the start, the book evokes that familiar sense of intellectual duelling between patient and clinician, with anorexia always at least one step ahead. This is one reason why I would recommend this book to any colleague with the stomach to read it, and to anyone who dares trivialise the challenge of working with people with eating disorders.

I found the spitefulness, the scorn, the superiority of anorexia quite chilling. It dehumanised Nancy, and made it hard to feel for her, although she suffered dreadfully. I laughed uncomfortably. I wondered with horror which of the three caricatured clinicians is me – Kind-face, Mean-face or Hangover face. Or, hopefully, ‘Sharp Psychiatrist’, even ‘The Right Therapist’. Patients have made me all of these at times. I cringed at the thought of her parents reading about their ‘marital(ish) bed’ – a quip which outlasts the apologies and appreciations at the end.

What did I learn? Well, that out-patient weights mean next to nothing. Nancy isn't unique in ‘faking’ as much as 10 kg – so why do we base clinical decisions on BMI alone? I knew that anorexia is like possession by devils but I saw it afresh. I learned from the black humour of Tucker's ‘questionnaires’ that there are no right answers. Whatever your responses to anorexia's dilemmas, you have got it wrong. That is the dynamic.

It's a chilling read, and both gratifying and irritating in turns. Tucker is a razor-sharp thinker and writer. The satirical dramatisations and caricatures evoke a stage adaptation – perhaps *Anorexia: The Musical*. As Tucker's style matures, her archness may be used more sparingly, she may edit out such distractions as colour titles and metaphors, and attend to the overall balance of the narrative, rather than merely acknowledge its imbalance. In fact, 300 pages give us the gory details of her anorexic career, while only the final 50 are devoted to the real meat of the story, the ‘time in between’ which the title evokes.

But I would not change the title. The last pages are the real genius of this book. The author poignantly describes her loss of anorexic power, descent into bulimia and weight gain. She re-enters real life, makes genuine relationships (including one with ‘the Right Therapist’) and experiences self-compassion for the first time. The spiteful start is transformed into lyrical, youthful, purple passages about her rites of passage. I was won over.

